# Impact of Ovariectomy on the Anterior Pituitary Gland in Female Rats

**DOI:** 10.1155/2023/3143347

**Published:** 2023-03-11

**Authors:** Aki Oride, Haruhiko Kanasaki, Tuvshintugs Tumurbaatar, Zolzaya Tumurgan, Hiroe Okada, Zhuoma Cairang, Kyo Satoru

**Affiliations:** Department of Obstetrics and Gynecology, Shimane University School of Medicine, Izumo, Shimane 693-8501, Japan

## Abstract

Ovariectomy (OVX) causes a depletion of circulating estradiol (E2) and influences hypothalamic kisspeptin neurons, which govern gonadotropin-releasing hormone (GnRH) release and ultimately gonadotropin secretion. In this study, we examined the changes induced by OVX on the anterior pituitary gland in female rats. OVX significantly increased the mRNA expression of gonadotropin *α*, luteinizing hormone (LH) *β*, and follicle-stimulating hormone (FSH) *β* subunits within the pituitary gland compared with control (sham-operated) rats, and this was completely suppressed by E2 supplementation. High-dose dihydrotestosterone supplementation also prevented the OVX-induced increase in the expression of the three gonadotropin subunits. GnRH receptor mRNA expression within the pituitary was significantly increased in OVX rats, and this increase was completely inhibited by E2 supplementation. The mRNA expression of the receptors for adenylate cyclase-activating polypeptide and kisspeptin was unchanged by OVX. Although the mRNA levels of inhibin *α*, *β*A, and *β*B subunits within the pituitary gland were not modulated by OVX, follistatin gene expression within the pituitary gland was increased by OVX, and this increase was completely inhibited by E2 supplementation after OVX. In experiments using a pituitary gonadotroph cell model (L*β*T2 cells), follistatin itself did not modulate the mRNA expression of gonadotropin LH*β* and FSH*β* subunits, and the GnRH-induced increase in the expression of these genes was slightly inhibited in the presence of follistatin. Our current observations suggest that OVX induces several characteristic changes in the pituitary gland of rats.

## 1. Introduction

The hypothalamic-pituitary-gonadal axis controls female reproductive function. At present, hypothalamic kisspeptin (encoded by the Kiss-1 gene) is believed to be the principal regulator of the hypothalamic-pituitary-gonadal axis [1]. Kisspeptin neurons are located in two areas of the hypothalamus and govern the different modes of gonadotropin-releasing hormone (GnRH) release. One is the surge secretion of GnRH, which is initiated at the late follicular phase with a concomitant increase in circulating estradiol (E2), which ultimately evokes a surge in the levels of the gonadotropin luteinizing hormone (LH) and induces ovulation. The second mode is the tonic release of GnRH, which maintains the pulsatile secretion of LH and follicle-stimulating hormone (FSH). In rodents, kisspeptin neurons located in the anteroventral periventricular nucleus are involved in the surge mode of GnRH release, while those located in the arcuate nucleus (ARC) control the tonic secretion of GnRH. The mechanisms underlying the regulation of the hypothalamic-pituitary-gonadal axis by kisspeptin neurons have been well characterized and reviewed widely [1–3].

E2 produced in the ovary plays an indispensable role in the maintenance of female reproductive function. In addition, E2 regulates the GnRH surge by a positive feedback mechanism and also regulates the tonic secretion of GnRH by a negative feedback mechanism. GnRH neurons lack E2 receptor *α* [4], but it is expressed by kisspeptin neurons [5, 6]. Furthermore, Kiss-1 gene expression is increased by E2 in the anteroventral periventricular nucleus, while its expression in the ARC is repressed by E2 [5, 6]. Therefore, kisspeptin neurons in the anteroventral periventricular nucleus and ARC are defined as sensors of E2 and induce positive and negative feedback control, respectively [1].

Gonadotropins in the anterior pituitary are under the control of hypothalamic kisspeptin and GnRH neurons, and the tonic release of GnRH, which is regulated by ARC kisspeptin neurons, regulates the pulsatile secretion of LH and FSH during the menstrual cycle by controlling the expression of the *β* subunit [7]. In addition to the signal from the hypothalamus, the plasticity of gonadotropins is also modulated by sex steroids released from the ovary. For example, E2 increases pituitary GnRH receptor (GnRHR) expression in several mammals, including rats [8, 9]. Testosterone also upregulates GnRHR expression in rats [10]. The expression of dopamine receptors in the pituitary gland is reported to be decreased by E2 in rats [11].

Although it is evident that gonadotropins are principally regulated by GnRH, the hypothalamic peptide pituitary adenylate cyclase-activating polypeptide (PACAP) also participates in the control of gonadotropin synthesis and release via the PACAP type I receptor (PAC1R) [12–14]. PACAP and PAC1R are thought to be involved in the differential regulation of LH and FSH by different frequencies of GnRH pulse stimulation [15]. In addition, the kisspeptin receptor (Kiss1R) is expressed in the pituitary gland and is involved in the synthesis of gonadotropins [16, 17]. Furthermore, activins, inhibins, and follistatin produced within the pituitary also participate in the control of gonadotropin synthesis and secretion. These peptides were originally identified from the ovarian follicular fluid as local factors that control follicular maturation and steroid synthesis [18], but activin, inhibin, and follistatin produced within the pituitary gland also act as autocrine and paracrine factors to regulate gonadotropin expression, especially FSH [19, 20].

In this study, we determined the local changes in the anterior pituitary induced by ovariectomy (OVX) using female rats. Specifically, we characterized the changes in gonadotropin subunit gene expression induced by OVX, the effects of sex steroid replacement, and the mRNA expression of hypothalamic peptide receptors, inhibin subunits, and follistatin within the anterior pituitary.

## 2. Materials and Methods

### 2.1. Materials

The following chemicals and reagents were obtained from the indicated sources: fetal bovine serum (Invitrogen, Carlsbad, CA); Dulbecco's modified Eagle's medium, water-soluble E2, dihydrotestosterone (DHT), follistatin, and penicillin-streptomycin (Sigma-Aldrich Co., St. Louis, MO); and 17*β*-E2 pellets (Innovative Research of America, Sarasota, FL).

### 2.2. Animal Experiments

Six-week-old female Wistar rats were maintained under a 12-h light/dark cycle at 20–25°C with food (CE-2; CLEA Japan, Tokyo, Japan) and water available *ad libitum*. Vaginal smears were taken daily, and only rats showing at least three consecutive regular estrus cycles were included in this study. Rats were ovariectomized under isoflurane anesthesia. Seven days later, a 17*β*-E2 (0.25 mg, 21-day release time) or placebo pellet was implanted subcutaneously. Then, the rats were bred for an additional period of 7 days. When we used DHT, a daily subcutaneous injection of DHT (5 or 25 mg/kg body weight/day) in 160 *μ*L sesame oil (Fujifilm, Tokyo, Japan) was given to the rats after OVX for 7 days. Sham-operated rats were used as controls. The rats were euthanized while under isoflurane anesthesia, and the anterior pituitary was removed for use in the experiments. This protocol was approved by the Ethics Committee of the Experimental Animal Center for Integrated Research at Shimane University (IZ31-51).

### 2.3. Cell Culture

L*β*T2 cells, a pituitary gonadotroph cell model, were plated on 35-mm tissue culture dishes and incubated in high-glucose Dulbecco's modified Eagle's medium containing 10% heat-inactivated fetal bovine serum and 1% penicillin-streptomycin at 37°C in a humidified atmosphere of 5% CO_2_ in the air. For stimulation, the cells were incubated with or without (control) test reagents at the indicated concentrations for 24 h in high-glucose Dulbecco's modified Eagle's medium containing 1% heat-inactivated fetal bovine serum and 1% penicillin-streptomycin.

### 2.4. RNA Preparation, Reverse Transcription, PCR, and Quantitative Real-Time PCR

Total RNA was extracted from rat pituitary tissue or L*β*T2 cells using TRIzol-LS (Invitrogen). To obtain cDNA, 1.0 *μ*g total RNA was reverse transcribed using an oligo-dT primer (Promega, Madison, WI) and prepared using a First-Strand cDNA Synthesis Kit (Invitrogen) and reverse transcription buffer. The preparation was supplemented with 10 mM dithiothreitol, 1 mM of each dNTP, and 200 U RNase inhibitor/human placenta ribonuclease inhibitor (Code No. 2310; Takara, Tokyo, Japan) in a final volume of 10 *μ*L. The reaction was incubated at 37°C for 60 min. The mRNA expression of gonadotropin *α*, LH*β*, and FSH*β* subunits, GnRHR, PAC1R, Kiss1R, inhibin *α*, *β*Α, and *βΒ* subunits, and follistatin was determined by quantitative real-time (RT)-PCR (ABI Prism 7000; Applied Biosystems, Foster City, CA) according to the manufacturer's instructions (User Bulletin No. 2) using Universal ProbeLibrary Probes and FastStart Master Mix (Roche Diagnostics, Mannheim, Germany). The PCR primers were designed based on published sequences of gonadotropin *α* [21], LH*β* and FSH*β* [22], GnRHR [23], PAC1R [24], Kiss1R [16], and inhibin *α*, *β*Α, and *βΒ* subunits and follistatin [25]. The simultaneous measurement of target mRNAs and GAPDH permitted the normalization of transcript levels. Each set of primers included a no-template control. The thermal cycling conditions were as follows: 10 min of denaturation at 95°C, followed by 40 cycles of 95°C for 15 s and 60°C for 1 min. Reactions were followed by a melting curve analysis (55°C–95°C). To determine PCR efficiency, 10-fold serial dilutions of cDNA were used as previously described [26]. PCR conditions were optimized to obtain >95% efficiency, and only those reactions with efficiencies between 95% and 105% were included in subsequent analyses. Relative differences in cDNA concentration between baseline and experimental conditions were calculated using the comparative threshold cycle (*Ct*) method [27]. Briefly, for each sample, Δ*Ct* was calculated for normalization against the internal control using the following equation: Δ*Ct* = Δ*Ct* (gene) − *Ct* (GAPDH). To obtain differences between experimental and control conditions, ΔΔ*Ct* was calculated as Δ*Ct* (sample) − Δ*Ct* (control). Relative mRNA levels were calculated using the following equation: fold difference = 2 − ΔΔ*Ct*.

### 2.5. Western Blot Analysis

Anterior primary tissues were homogenized on ice with RIPA buffer (phosphate-buffered saline, 1% NP-40, 0.5% sodium deoxycholate, and 0.1% sodium dodecyl sulfate [SDS]) containing 0.1 mg/mL phenylmethyl sulfonyl fluoride, 30 mg/mL aprotinin, and 1 mM sodium orthovanadate and centrifuged at 14,000×g for 10 min at 4°C. Protein concentration in the lysates was measured using the Bradford method. Denatured protein (30 *μ*g protein) was resolved by 10% SDS-polyacrylamide gel electrophoresis (PAGE) according to the standard protocols. Protein was transferred onto polyvinylidene difluoride membranes (Hybond-P PVDF; Amersham Biosciences, Little Chalfont, UK), which were blocked for 2 h at room temperature in Blotto (5% milk in Tris-buffered saline). The membranes were incubated with a monoclonal anti-rabbit FSH antibody (cat. no. ab180489) (1 : 1,000 dilution; Abcam, Cambridge, MA) in Blotto overnight at 4°C and washed three times for 10 min per wash with Tris-buffered saline/1% Tween 20. Subsequent incubation with mouse anti-rabbit horseradish peroxidase (HRP)-conjugated antibody (cat. no. sc-2357) (1 : 1,000 dilution; Santa Cruz Biotechnology, Santa Cruz, CA) was performed for 1 h at room temperature in Blotto, and additional washes were performed as needed. Following enhanced chemiluminescence detection (Amersham Biosciences), the membranes were exposed to X-ray film (Fujifilm, Tokyo, Japan). After strip washing (Restore Buffer; Pierce Chemical Co., Rockford, IL), the membrane was reprobed with monoclonal mouse anti-*β* actin primary antibody (cat. no. ab6276) (1 : 5,000 dilution; Abcam, Cambridge, MA) and mouse anti-rabbit IgG-HRP-conjugated secondary antibody (cat. no. sc-2357) (1 : 20,000 dilution; Jackson ImmunoResearch Labs Inc., Philadelphia, PA) and then visualized. To compare the levels of FSH, films were analyzed by densitometry, and the intensities of the bands were normalized to those of *β*-actin to correct for protein loading.

### 2.6. Reporter Plasmids and Luciferase Assay

Reporter plasmids that were generated by fusing −797/+5 of the rat LH*β* gene (LH*β*-Luc) or −2,000/+698 of the rat FSH*β* gene (FSH*β*-Luc) to firefly luciferase cDNA in pXP2 were generously provided by Dr. Ursula Kaiser [28, 29]. The cells were transiently transfected via electroporation together with 2.0 *μ*g/well gonadotropin subunit-Luc and 0.1 *μ*g pRL-TK (Promega), which expresses Renilla luciferase. After incubation with GnRH (100 nM), follistatin (10 ng/mL), or GnRH + follistatin for 6 h, the cells were washed with ice-coldphosphate-buffered saline and lysed with Passive Lysis Buffer (Promega). After centrifugation at 15,000 rpm at 4°C, firefly luciferase and Renilla luciferase activity in the supernatant was measured using the Dual-Luciferase Reporter Assay System and a TD-20/20 luminometer (both from Promega). Firefly luciferase activity was normalized to that of Renilla luciferase to correct for transfection efficiency, and the results were expressed as the level (fold) of increase compared with the unstimulated control.

### 2.7. Statistical Analysis

All experiments were repeated at least three times independently. Each experiment in each experimental group was performed using duplicate samples. When we determined mRNA expression, two samples were assayed in duplicate. Six averages from three independent experiments were statistically analyzed. Data are expressed as the mean ± standard error of the mean (SEM). Statistical analysis was performed using one-way analysis of variance with Bonferroni's post hoc test or Student's *t*-test as appropriate. *P* < 0.05 was considered statistically significant.

## 3. Results

### 3.1. Effect of OVX and E2 Supplementation on Gonadotropin Subunit Gene Expression

**Figure 1:**
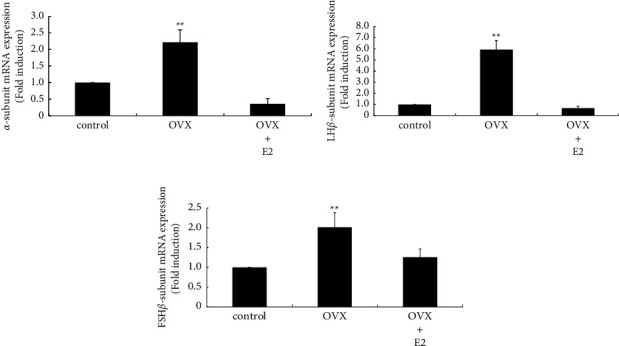
Effect of E2 supplementation on gonadotropin subunit gene expression after OVX. Gonadotropin *α* (a), LH*β* (b), and FSH*β* subunit (c) mRNA expression from the anterior pituitary in sham-operated (control), OVX, and OVX + E2 rats was compared by quantitative RT-PCR analysis. Samples for each experimental group were run in duplicate and normalized to the mRNA levels of GAPDH as a housekeeping gene. The results are expressed as fold induction over control and presented as the mean ± SEM. ^*∗∗*^*P* < 0.01 vs. control.

The impact of OVX on the mRNA expression of pituitary gonadotropin subunits was examined. As expected, the expression of all three gonadotropin subunits was significantly increased by OVX. Gonadotropin *α* subunit gene expression was increased by 2.22 ± 0.37-fold after OVX, and this increase was completely inhibited by E2 supplementation after OVX (Figure 1(a)). OVX also dramatically increased LH*β* subunit gene expression by 5.93 ± 0.82-fold (Figure 1(b)). Similarly, FSH*β* subunit gene expression was increased after OVX by 2.01 ± 0.37-fold (Figure 1(c)). The increase in LH*β* and FSH*β* subunit gene expression was completely inhibited by E2 supplementation after OVX.

### 3.2. Effect of OVX and DHT Supplementation on Gonadotropin Subunit Gene Expression

**Figure 2:**
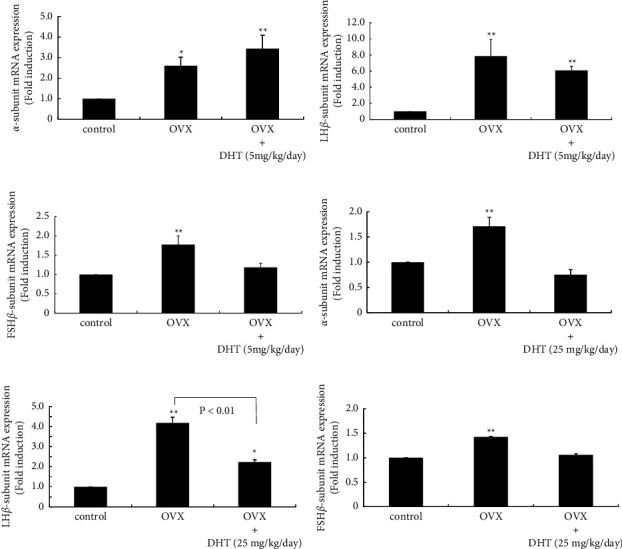
Effect of DHT supplementation on gonadotropin subunit gene expression after OVX. Gonadotropin *α* (a, d), LH*β* (b, e), and FSH*β* subunit (c, f) mRNA expression from the anterior pituitary in sham-operated (control), OVX, OVX + DHT (5 mg/kg body weight/day) (a, b, and c), and OVX + DHT (25 mg/kg body weight/day) (d, e, and f) rats were compared by quantitative RT-PCR analysis. Samples for each experimental group were run in duplicate and normalized to the mRNA levels of GAPDH as a housekeeping gene. The results are expressed as fold induction over control and presented as the mean ± SEM. ^*∗∗*^*P* < 0.01, ^*∗*^*P* < 0.05 vs. control. LH*β* subunit gene expression E differed significantly (*P* < 0.01) between OVX and OVX + DHT (25 mg/kg body weight/day) rats.

Next, we examined the effect of DHT supplementation after OVX on gonadotropin subunit gene expression. The mRNA expression of all three gonadotropin subunits was increased after OVX. DHT supplementation (5 mg/kg body weight/day) after OVX did not prevent the increase in gonadotropin *α* and LH*β* subunit gene expression (Figures 2(a) and 2(b)); however, the same dose of DHT supplementation inhibited the significant increase in FSH*β* subunit gene expression by OVX (Figure 2(c)). When a much higher dose of DHT was administered to the rats after OVX, DHT prevented the increased expression of all three gonadotropin subunit genes. The OVX-induced increase of gonadotropin *α* and FSH*β* subunit gene expression was completely inhibited by 25 mg/kg body weight/day DHT supplementation (Figures 2(d) and 2(f)). The OVX-induced increase in LH*β* subunit gene expression was also prevented by supplementation with a high dose of DHT (25 mg/kg body weight/day) (Figure 2(e)).

### 3.3. Effect of OVX and Sex Steroid Supplementation on FSH Protein Expression in the Anterior Pituitary

**Figure 3:**
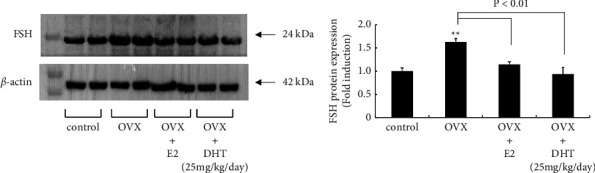
Effect of E2 and high-dose DHT supplementation on gonadotropin subunit gene expression after OVX. (a) Cell lysates (30 *μ*g protein) from the anterior pituitary in sham-operated (control), OVX, OVX + E2, and OVX + DHT (25 mg/kg body weight/day) rats were analyzed by SDS-PAGE followed by immunoblotting and incubation with antibody against FSH. *β*-actin was detected as an internal control. The bands were visualized using HRP-conjugated secondary antibody. (b) Scanning densitometry of the visualized bands using NIH ImageJ software was performed to determine differences in protein expression, normalized to that of *β*-actin. ^*∗∗*^*P* < 0.01 vs. control. FSH protein expression (E) differed significantly (*P* < 0.01) between OVX and OVX + E2 and OVX + DHT (25 mg/kg body weight/day) rats.

To confirm the effect of OVX and sex steroid supplementation on pituitary gonadotropin hormone, FSH expression within the anterior pituitary was determined at the protein level. OVX significantly increased FSH expression within the anterior pituitary by 1.63 ± 0.07-fold. This increase was completely prevented by E2 and high-dose DHT supplementation (Figures 3(a) and 3(b)).

### 3.4. Effect of OVX on the mRNA Expression of GnRH, PACAP, and Kisspeptin Receptors in the Anterior Pituitary

**Figure 4:**
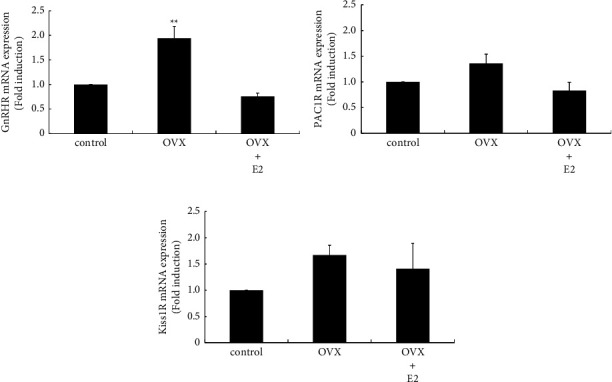
Changes in receptor expression after OVX. GnRHR (a), PAC1R (b), and Kiss1R (c) mRNA expression from the anterior pituitary in sham-operated (control), OVX, and OVX + E2 rats were compared by quantitative RT-PCR analysis. Samples for each experimental group were run in duplicate and normalized to the mRNA levels of GAPDH as a housekeeping gene. The results are expressed as fold induction over control and presented as the mean ± SEM. ^*∗∗*^*P* < 0.01 vs. control.

To examine whether the expression of the receptors for secretagogues of gonadotropins was changed by OVX, the mRNA levels of GnRH, PACAP, and kisspeptin receptors within the anterior pituitary were determined. OVX significantly increased GnRHR gene expression by 1.94 ± 0.25-fold, which was completely inhibited by E2 supplementation after OVX (Figure 4(a)). However, PAC1R and Kiss1R gene expression was not modulated by OVX (Figures 4(b) and 4(c)).

### 3.5. Effect of E2 on Gonadotropin Subunit Gene Expression in Pituitary Gonadotroph L*β*T2 Cells

**Figure 5:**
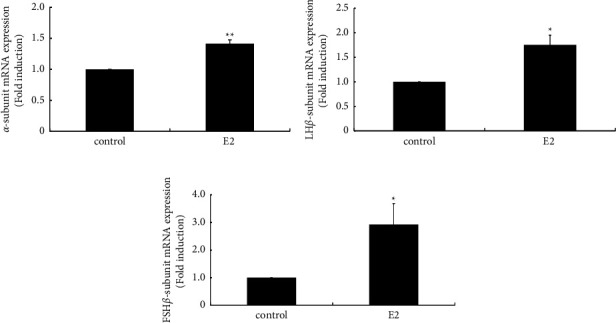
Effect of E2 on gonadotropin subunit gene expression in L*β*T2 cells. L*β*T2 cells were stimulated with 10 nM E2 for 24 h after which mRNA was extracted and reverse transcribed. Gonadotropin *α* (a), LH*β* (b), and FSH*β* subunit (c) mRNA levels were measured by quantitative RT-PCR. Samples for each experimental group were run in duplicate and normalized to the mRNA levels of GAPDH as a housekeeping gene. The results are expressed as fold induction over control and presented as the mean ± SEM. ^*∗∗*^*P* < 0.01, ^*∗*^*P* < 0.05 vs. control.

Because the mRNA expression of the three gonadotropin subunits was significantly increased by depletion of E2 after OVX in rats, we next examined the effect of E2 on gonadotropin subunit gene expression using pituitary gonadotroph L*β*T2 cells. E2 (10 nM) significantly increased the expression of the *α* subunit by 1.41 ± 0.06-fold (Figure 5(a)), LH*β* subunit by 1.76 ± 0.20-fold (Figure 5(b)), and FSH*β* subunit by 2.92 ± 0.76-fold (Figure 5(c)).

### 3.6. Effect of OVX on the mRNA Expression of Inhibin Subunits and Follistatin within the Pituitary Gland

**Figure 6:**
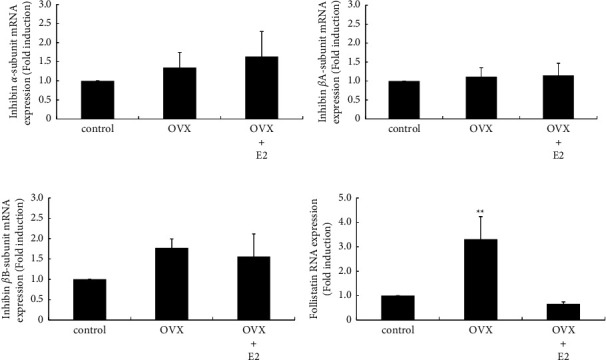
Changes in inhibin subunit expression after OVX. Inhibin *α* (a), *β*Α (b), and *βΒ* (c) and follistatin (d) mRNA expression from the anterior pituitary in sham-operated (control), OVX, and OVX + E2 rats was compared by quantitative RT-PCR analysis. Samples for each experimental group were run in duplicate and normalized to the mRNA levels of GAPDH as a housekeeping gene. The results are expressed as fold induction over control and presented as the mean ± SEM. ^*∗∗*^*P* < 0.01 vs. control.

The activin/inhibin/follistatin system participates in the regulation of gonadotropins, especially FSH [19, 20, 30]. Therefore, we examined the mRNA expression of inhibin subunits, which compose activin or inhibin, and follistatin within the anterior pituitary. Inhibin *α* subunit gene expression was not modulated by OVX (Figure 6(a)). Similarly, neither inhibin *β*A nor *β*B subunit gene expression was changed within the pituitary gland by OVX (Figures 6(b) and 6(c)). Conversely, follistatin gene expression was significantly increased in the pituitary gland after OVX by 3.31 ± 0.93-fold, which was completely inhibited by E2 supplementation after OVX (Figure 6(d)).

### 3.7. Effect of Follistatin on GnRH-Induced Gonadotropin Subunit Gene Expression

**Figure 7:**
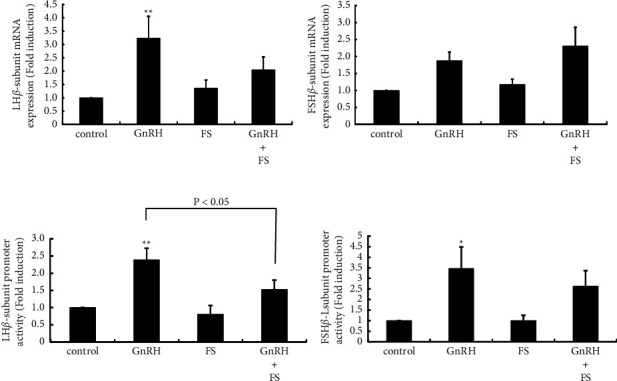
Effect of follistatin on GnRH-induced gonadotropin subunit expression (a, b). L*β*T2 cells were stimulated with 100 nM GnRH, 10 ng/mL follistatin (FS), or both for 24 h. Then, gonadotropin LH*β* (a) and FSH*β* (b) subunit mRNA levels were measured by quantitative RT-PCR. Samples for each experimental group were run in duplicate and normalized to the mRNA levels of GAPDH as a housekeeping gene. (c and d) L*β*T2 cells were cotransfected with pRL-TK (0.1 *μ*g) plus 2.0 *μ*g LH*β*-Luc (c) or FSH*β*-Luc (d) vectors. At 48 h after transfection, the cells were treated with 100 nM GnRH, 10 ng/mL FS, or both for 6 h. A luciferase assay was performed to examine LH*β* and FSH*β* promoter activity, which was normalized to Renilla luciferase activity. The results are expressed as fold induction over control and presented as the mean ± SEM. ^*∗∗*^*P* < 0.01, ^*∗*^*P* < 0.05 vs. control.

The effect of follistatin on GnRH-induced gonadotropin LH*β* and FSH*β* subunit gene expression was examined using L*β*T2 cells. GnRH significantly increased LH*β* subunit mRNA expression by 3.23 ± 0.83-fold. Follistatin itself had no effect on the expression of LH*β* mRNA. However, the GnRH-induced increase in LH*β* subunit mRNA was blunted in the presence of follistatin (Figure 7(a)). Follistatin did not change the basal activity of the LH*β* promoter, but GnRH-induced LH*β* promoter activity was significantly inhibited in the presence of follistatin (Figure 7(c)). Although we did not observe a significant effect of GnRH on FSH*β* mRNA expression by quantitative RT-PCR analysis, GnRH increased FSH*β* mRNA expression by 1.87 ± 0.26-fold compared with nonstimulated cells. It was not statistically significant, but GnRH plus follistatin increased FSH*β* mRNA expression by 2.31 ± 0.55-fold (Figure 7(b)). Similarly, the GnRH-induced increase of FSH*β* promoter activity was not significantly inhibited in the presence of follistatin, although a significant increase was not obtained by GnRH plus follistatin stimulation (Figure 7(d)). Follistatin itself did not change the gene expression or promoter activity of the FSH*β* subunit (Figures 7(b) and 7(d)).

## 4. Discussion

The serum levels of gonadotropins gradually increase in older women because the E2-induced negative feedback mechanism is disrupted by the failure of ovarian function with a concomitant decrease in E2 secretion. Similarly, the artificial depletion of E2 by OVX induces a sudden elevation of gonadotropins by the same negative feedback mechanism. In this study, we examined the effect of E2 depletion on the anterior pituitary gland following OVX. The expression of three gonadotropin subunits, receptors for hypothalamic factors, and inhibin subunits were determined after OVX.

As for the expression of the gonadotropin common *α* subunit and specific LH*β* and FSH*β* subunits, it was increased by OVX as a result of the negative feedback mechanism. Because E2 supplementation after OVX completely prevented the increased expression of all gonadotropin subunits, the OVX-induced increase in their expression could be explained by the depletion of E2. At present, it is generally agreed that kisspeptin neurons located in the ARC region of the hypothalamus govern the pulsatile tonic release of GnRH [1, 2]. Kiss-1 is highly expressed in the ARC at diestrus, while it is suppressed in OVX mice by E2 treatment [5, 31]. Furthermore, the E2-dependent modification of histone H3 in the Kiss-1 promoter region in the mouse ARC results in the repression of Kiss-1 expression [32]. Therefore, we could speculate that the OVX-induced increase in the expression of all three gonadotropin subunits was dependent on the increase in the Kiss-1 gene and kisspeptin expression in the ARC. E2 supplementation after OVX prevented the increase of gonadotropin subunit expression because it inhibited the increase of kisspeptin expression in this hypothalamic area. Supplementation with a high dose of the androgen DHT also prevented the increase in the expression of the three gonadotropin subunits in OVX rats, suggesting that the OVX-dependent increase in Kiss-1 and kisspeptin expression might also be repressed by DHT. Kisspeptin neurons in the ARC (also called KNDy neurons because they coexpress kisspeptin, neurokinin B, and dynorphin A) [33] express estrogen and androgen receptors [34]. Unlike the effect of E2 on KNDy neurons in the ARC, a previous report showed that DHT treatment increases the kisspeptin signal in the ARC of rats [35]. However, in the present study, the significant increase in the expression of the three gonadotropin subunit genes induced by OVX was prevented by DHT as well as E2 supplementation. Because the increased mRNA expression of gonadotropin *α* and LH*β* was not prevented by low-dose DHT supplementation, it seems that a sufficient concentration of DHT is necessary to affect kisspeptin neurons and repress their functions. Indeed, androgens are reported to have a feedback effect on KNDy neurons in the ARC region of the hypothalamus. Smith et al. demonstrated that the castration-induced increase in Kiss-1 gene expression in the ARC hypothalamus of male mice was prevented by testosterone. Furthermore, they revealed that the reversible effect of testosterone on ARC Kiss-1 gene expression was mediated via androgen receptors and via estrogen receptors after testosterone was aromatized to estrogen. Because DHT is a nonaromatizable androgen, its effect might be weak compared with testosterone. Therefore, a low dose of DHT could not prevent the increase in the expression of all three gonadotropin subunits after OVX.

Depletion of E2 by OVX induced a drastic increase in the gene expression of all three gonadotropin subunits; however, E2 itself stimulated their expression in pituitary gonadotroph L*β*T2 cells. These observations suggest that gonadotropins are under the influence of E2 and the three gonadotropin subunits could be upregulated by E2; however, considering the observation that all three gonadotropin subunits were increased by OVX, it is plausible that E2 has an insignificant effect on gonadotropins. Rather, hypothalamic factors such as GnRH and kisspeptin are principal regulators of gonadotropin subunit gene expression.

Among the receptors for hypothalamic factors expressed in the anterior pituitary, the GnRHR gene was upregulated by OVX, and this increase was repressed by E2 supplementation after OVX. These changes were comparable to those observed for the mRNA expression of the three gonadotropin subunits. Pituitary hormone-secreting cells as well as folliculostellate cells express the receptor for PACAP, PAC1R [36], and gonadotropins that are clearly regulated by PACAP with direct effects on gonadotropin secretion and subunit gene expression [13, 37]. However, PAC1R expression was not influenced by E2 depletion. These observations imply that the changes in the expression of receptors for hypothalamic factors are also principally regulated by the changes in the secretory pattern of GnRH, which is governed by hypothalamic kisspeptin. The GnRH pulse frequency-dependent regulation of gonadotropin *β* subunit gene expression is well established. That is, more rapid GnRH pulses increase the gene expression and secretion of LH, whereas slower frequencies increase the mRNA expression and secretion of FSH [7, 38]. In addition, a previous study demonstrated that GnRH itself regulates GnRHR expression, and its expression is increased further at higher GnRH pulse frequencies [39]. Because OVX induces a higher frequency of GnRH pulse secretion by increasing kisspeptin expression in KNDy neurons in the ARC, increased GnRHR expression in the anterior pituitary might be induced by higher frequencies of GnRH pulse secretion after OVX. In contrast, although PAC1R expression is also regulated by GnRH, lower frequencies of GnRH pulse stimulation tend to stimulate PAC1R expression [40]. Therefore, it is plausible that higher frequencies of GnRH pulsatile secretion after OVX did not increase PAC1R expression within the anterior pituitary. Although it is evident that kisspeptin and its receptor (Kiss1R) are expressed in the pituitary gland and are under the control of GnRH [17, 41], OVX did not increase Kiss1R expression. Because there is no doubt that GnRH secretion is increased by OVX, the absence of an increase in Kiss1R gene expression after OVX might be caused by its higher pulse frequency.

We also determined the changes in inhibin subunit expression within the anterior pituitary because the activin/inhibin/follistatin system works locally within the pituitary gland and regulates FSH [19, 20, 30]. The mRNA expression of the inhibin *α*, *β*A, and *β*B subunits was unaltered by OVX, indicating that the local expression of activins and inhibins in the pituitary gland might not be dramatically changed by OVX. In contrast, follistatin gene expression in the pituitary gland was significantly increased by OVX, and this increase was completely inhibited by E2 supplementation after OVX. This result was quite similar to those observed for the gonadotropin subunits and GnRHR. Indeed, previous studies reported that follistatin gene expression is increased only following stimulation with high-frequency GnRH pulses [42, 43]. Thus, we suspected that the changes in follistatin gene expression induced by OVX might be caused by the altered secretion of GnRH from the hypothalamus. In the experiments using L*β*T2 cells, follistatin did not produce a striking effect on gonadotropin subunit expression; however, the presence of follistatin seemed to inhibit GnRH-induced LH*β* and FSH*β* subunit gene expression and promoter activity slightly. Nevertheless, these phenomena were very modest, and it remains unclear whether follistatin has an effect on the action of GnRH. If follistatin has an inhibitory effect on GnRH-induced gonadotropin synthesis, it might exert some inhibitory effects on the elevation of gonadotropins after OVX.

## 5. Conclusion

In this study, we examined the local changes in the anterior pituitary after OVX in female rats. As expected, the expression of all three gonadotropin subunit genes was increased by OVX, and these increases were inhibited by E2 supplementation after OVX. High-dose DHT supplementation also prevented the increase in the mRNA expression of all three gonadotropin subunits after OVX. In addition to the three gonadotropin subunits, GnRHR and follistatin gene expression was also increased in the pituitary gland by OVX, and these increases were suppressed by E2 supplementation. It is plausible that the changes in the expression of these genes were the result of the high frequency of GnRH pulse secretion after OVX.

## Data Availability

The datasets used and/or analyzed during this study are available from the corresponding author upon reasonable request.
